# Histone H3 Lysine 9 Acetylation is Downregulated in GDM Placentas and Calcitriol Supplementation Enhanced This Effect

**DOI:** 10.3390/ijms19124061

**Published:** 2018-12-14

**Authors:** Paula Hepp, Stefan Hutter, Julia Knabl, Simone Hofmann, Christina Kuhn, Sven Mahner, Udo Jeschke

**Affiliations:** 1 Department of Gynecology and Obstetrics, University Hospital, LMU Munich, Maistraße 11, 80337 Munich, Germany; paula.h@gmx.de (P.H.); hutter.stefan@googlemail.com (S.H.); julia.knabl@gmx.de (J.K.); simone.hofmann@med.uni-muenchen.de (S.H.); Christina.kuhn@med.uni-muenchen.de (C.K.); sven.mahner@med.uni-muenchen.de (S.M.); 2 Department of Obstetrics, Klinik Hallerwiese, 90419 Nürnberg, Germany

**Keywords:** histone modification, H3K9ac, H3K4me3, FOXO1, epigenetics, gestational diabetes, vitamin D

## Abstract

Despite the ever-rising incidence of Gestational Diabetes Mellitus (GDM) and its implications for long-term health of mothers and offspring, the underlying molecular mechanisms remain to be elucidated. To contribute to this, the present study’s objectives are to conduct a sex-specific analysis of active histone modifications in placentas affected by GDM and to investigate the effect of calcitriol on trophoblast cell’s transcriptional status. The expression of Histone H3 lysine 9 acetylation (H3K9ac) and Histone H3 lysine 4 trimethylation (H3K4me3) was evaluated in 40 control and 40 GDM (20 male and 20 female each) placentas using immunohistochemistry and immunofluorescence. The choriocarcinoma cell line BeWo and primary human villous trophoblast cells were treated with calcitriol (48 h). Thereafter, western blots were used to quantify concentrations of H3K9ac and the transcription factor FOXO1. H3K9ac expression was downregulated in GDM placentas, while H3K4me3 expression was not significantly different. Cell culture experiments showed a slight downregulation of H3K9ac after calcitriol stimulation at the highest concentration. FOXO1 expression showed a dose-dependent increase. Our data supports previous research suggesting that epigenetic dysregulations play a key role in gestational diabetes mellitus. Insufficient transcriptional activity may be part of its pathophysiology and this cannot be rescued by calcitriol.

## 1. Introduction

Gestational Diabetes Mellitus (GDM) is defined as glucose intolerance firstly detected during pregnancy [1]. It is the most common pregnancy-related metabolic disorder, affecting up to 14% of pregnancies [2]. Contrary to other obstetric complications, its prevalence has increased steadily over the past decades, making it an urgent issue in pre- and perinatal care [3,4].

The short-term consequences of GDM include a higher risk for preeclampsia (Odds Ratio (OR) 1.81), large for gestational age infants (LGA) (OR 3.43) and C-section (OR 1.46) [5,6]. However, GDM also affects long-term health outcomes of both mothers and offspring. Women who suffered from GDM stand a substantially higher risk of developing diabetes mellitus type 2 (DM2) later in life (relative risk (RR) 7.43) [7]. Their offspring also suffer from a higher risk for developing metabolic syndrome, DM2, adiposity and cardiovascular disease [8,9,10].

GDM manifests when the increased need for insulin during pregnancy cannot be met, leading to a state of glucose intolerance, insulin resistance and inflammation similar to DM2 [11,12]. As the producer of anti-insulin hormones and in its function as the foeto-maternal-interface, the placenta is thought to play a key role in the pathophysiology of GDM in general and, more specifically, in the long-term health programming of the offspring [13,14]. However, despite the urgent need for effective treatment and disease prevention in future generations, the pathophysiology’s underlying molecular mechanisms are not yet fully understood.

Increasingly, pathophysiological research focuses on transcriptional and epigenetic mechanisms of insulin resistance. Evidence constantly emerges that transcription factors, including FOXO1, co-factors and chromatin modifications, like post-translational histone modifications, interact on various levels of gene expression and through this, contribute to insulin resistance. These mechanisms may also be part of the developmental programming of metabolic diseases [15].

Barker’s “Developmental Origins of Human Health and Disease” theory, stating that foetal environment influences health outcomes in later life, first arose from investigations of the relationship between birthweight and cardiovascular diseases in adulthood [16,17]. Since then, results from various cell culture and animal models, as well as epidemiological investigations point towards epigenetics as being one of the underlying molecular mechanisms [18,19,20,21].

Epigenetics can be seen as “the sum of the alterations to the chromatin template that collectively establish and propagate different patterns of gene expression and silencing from the same genome” [22]. The three main mechanisms interacting to form the epigenome are: DNA methylation, histone modifications and non-coding RNA (ncRNA) [22]. While evidence of epigenetic changes in GDM cases is growing, research has been focusing mainly on DNA methylation studies, with data on histone modifications in GDM is still widely lacking [23].

Post-translational Modification (PTM) of histone proteins influences transcription, DNA replication and other processes via cis and/or trans mechanisms. The direct cis mechanism describes a change of inter-nucleosome contact, thus affecting the chromatin condensation state. Histone acetylation is known to have a vast potency for unfolding chromatin. The indirect trans mechanism refers to the recruitment of “reader” proteins to the chromatin, which in turn recruit further modifying proteins. Additionally, to cis and trans interactions, interaction between different PTMs, so called “cross-talk”, also occur [22,24,25]. As the “cross-talk” interactions indicate, the sum of histone modifications results in a histone-code, which determines the accessibility of a given chromatin section.

The two modifications acetylation of histone 3 at lysine 9 (H3K9ac) and trimethylation of histone 3 at lysine 4 (H3K4me3) are known to be “active” modifications, and their presence at Transcriptional Start Site (TSS) regions correlates with the amplitude of gene expression [26]. High levels of H3K4me3 can be found specifically at the promotor region of active genes, because it plays a role in forming the preinitiation complex [27,28]. H3K9ac enrichment, on the other hand, marks active enhancers. Furthermore, both PTMs are found at bivalent promoters marking regulatory genes of importance for cell differentiation [26,29]. These findings indicate that both H3K9ac and H3K4me3, like epigenetics in general, are of great importance for an organism’s development and human disease. Their role in GDM, however, has not yet been investigated. 

FOXO1 is part of the Forkhead box protein subfamily O, a group of transcription factors containing winged helix DNA-binding domain [30]. In its function as a transcription factor, it plays a key role in metabolic regulation, especially in the glucose metabolism. Increased FOXO1 has been shown to stimulate gluconeogenesis as well as lipid production in liver cells [31,32]. Reduced FOXO1 function on the other hand was shown to protect against insulin resistance under a high fat diet in mice [33]. More recent results were able to show that FOXO1 is elevated in GDM placentas on mRNA as well protein level. Furthermore, placental *FOXO1* mRNA expression was significantly correlated with the HOMA-IR (Homeostatic Model Assessment of Insulin Resistance) in the GDM group [34]. Further studies with vitamin D receptor (VDR) knockout mice extrapolated a functional link between vitamin D deficiency, elevated FOXO1 and insulin resistance in muscle cells [35]. 

The epidemiological connection between vitamin D deficiency and GDM has been discussed, but remains controversial, with several studies suggesting that there is a positive correlation between low vitamin D and insulin resistance or GDM respectively [36,37,38], while others did not find a significant connection [39,40,41]. Recent metanalyses suggest, however, that vitamin D deficiency is in fact a risk factor (OR) for the development of GDM [42,43]. Investigations on GDM placentas have shown an increased expression of VDR compared to control placentas and hypothesised this to be a reaction to low vitamin D levels [44]. Recently, ChiP-seq analyses in cell culture experiments by Meyer, et al. [45] showed that stimulation with Calcitriol (1,25(OH)_2_D_3_) lead to an enrichment of H3K9ac at promotor regions of VDR regulated genes, suggesting a link between epigenetic modifications and vitamin D.

The aim of this study was to conduct a systematic, sex-specific immunohistochemical and immunofluorescence analysis of active histone modifications in GDM placentas. Furthermore, cell culture experiments were carried out to test the hypothesis that vitamin D stimulation could increase H3K9ac and correspondingly decrease FOXO1 expression in the choriocarcinoma cell line BeWo. Cell culture results were verified using primary human villous trophoblast (HVT) cultures.

We found H3K9ac to be upregulated in syncytiotrophoblast, extra villous trophoblast (EVT), as well as foetal endothelial cells in GDM placentas. The analysis of H3K4me3 did not reveal any significant differences in expression between GDM and control placentas. The stimulation of BeWo cells with human calcitriol resulted in a decrease of H3K9ac at high concentrations and no significant changes at low concentrations. This corresponded with an increase in FOXO1 expression after stimulation. This indicates that the dysregulated H3K9ac expression in GDM cannot be salvaged by vitamin D.

## 2. Results

The expression of specific post-translational modifications of histone protein 3 were analysed in placental tissue from 40 heathy (20 of female, 20 of male foetuses) and 40 GDM pregnancies (20 of female, 20 of male foetuses) using immunohistochemistry and double immunofluorescence. The modifications under investigation were H3 lysine 9 acetylation (H3K9ac) and H3 lysine 4 trimethylation (H3K4me3).

### 2.1. H3K9ac Expression is Downregulated in GDM Placentas

**Figure 1:**
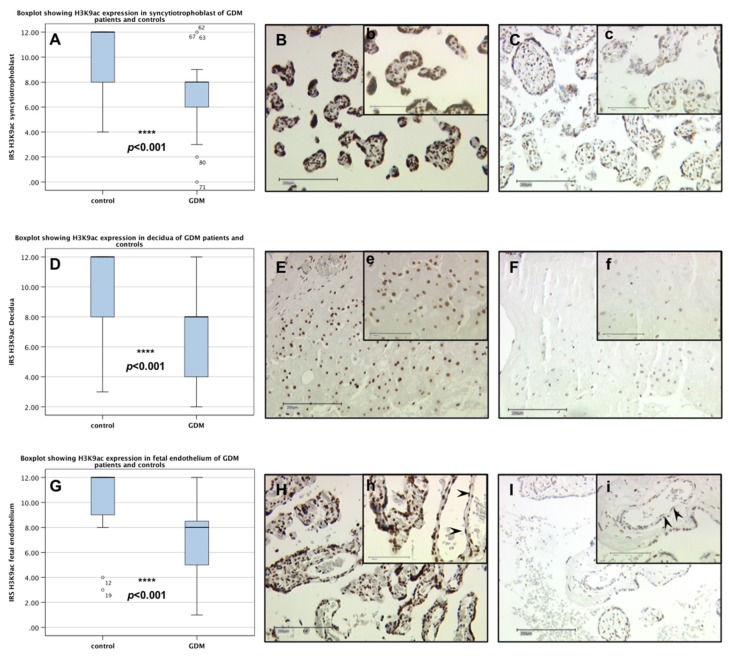
H3K9ac expression in syncytiotrophoblast, decidua and foetal endothelia cells of control and GDM placentas. Boxplots (**A**,**D**,**G**) showing the IRS for H3K9ac expression in syncytiotrophoblast (SCT), decidua and foetal endothelial to be highly significantly lower in GDM placentas (*p* < 0.001). The range between the 25th and 75th percentiles is represented by the boxes with the horizontal line showing median. The bars indicate the 5th and 95th percentiles. Circles indicate values more than 1.5-times the boxes’ lengths. Pictures showing representative slides for immunohistochemical staining of H3K9ac in the SCT (control (**B**,**b**); GDM (**C**,**c**)), decidua (control (**E**,**e**); GDM (**F**,**f**)) and foetal endothelial cells (control (**H**,**h**); GDM (**I**,**i**)) of according placentas. Arrowheads indicate foetal endothelial cells. Pictures were taken with a 100× lens (capital letters) and 250× lens (lower-case letters) respectively.

There was no sex-specific difference of H3K9ac expression within the control group, nor within the GDM group. Therefore, we grouped male and female data of control and GDM group for the following data analysis. A very strong H3K9ac expression (median immuno reactive score (IRS): 12) was detected in nuclei of villous syncytiothrophoblast cells (SCT), extra villous trophoblast cells (EVT) as well as foetal endothelial cells in control placentas. The boxplot in Figure 1A illustrates that the expression of H3K9ac was significantly downregulated in the syncytiotrophoblast cells of GDM placentas (*p* < 0.001), with a median IRS of 12 in the control compared to 8 in the GDM placentas. The same pattern was found in EVTs (Figure 1D–F) and foetal endothelial cells (Figure 1G–I), where statistical analysis proved the downregulation to be highly significant (*p* < 0.001). 

### 2.2. No Difference between H3K4me3 Expression in GDM and Control Placentas

In contrast to H3K9ac expression, no statistically significant differences were found in the expression of H3K4me3 (see Figure S1). The median IRS for H3K4me3 expression in the syncytiotrophoblast was 8 in control, as well as GDM placentas (*p* = 0.853). Similar results were obtained for EVTs. There was however a significant difference in the H3K4me3 expression between male and female control placentas (*p* = 0.040).

### 2.3. Identification of H3K9ac Expressing Cells by Immunofluorescence Double Staining

**Figure 2:**
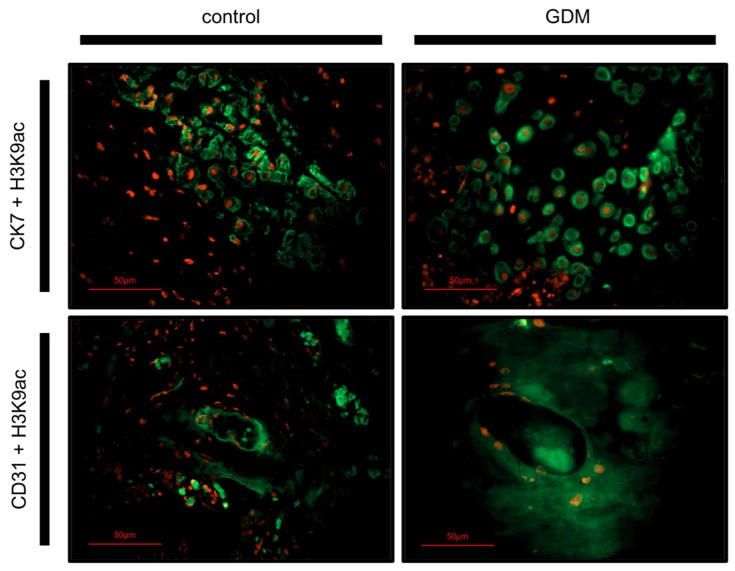
Double immunofluorescence phenotyping of placenta cells. H3K9ac, marked with Cy-3-labled secondary antibody, is stained red in both rows. CK7 is stained green in the first row, marking EVT cells. CD31 is stained green in the second row, marking endothelial cells. Pictures were taken with a 400× lens.

Immunofluorescence double staining was carried out using Cytokeratin 7 (CK7) and Cluster of differentiation 31 (CD31) as identifying markers for EVTs and foetal endothelial cells respectively. By using triple filter excitation microscopy, cell phenotypes could be identified (Figure 2).

Double filter excitation showed CK7 and H3K9ac expression in the same cell, thus confirming the expression of H3K9ac by EVTs. H3K9ac was also expressed by some CK7-negative decidual stroma cells. The H3K9ac expression was more intense in EVT cells of control placentas than GDM placentas. Similarly, double filter excitation showed co-expression of CD31 and H3K9ac, confirming H3K9ac expression by endothelial cells. Again, the H3K9ac expression was more intense in endothelial cells of control placentas than GDM placentas.

### 2.4. Downregulation of H3K9ac in Trophoblast Tumour Cells BeWo by Human Calcitriol (Vitamin D)

To gain insight into the possible functional relationship between H3K9ac and FOXO1 expression and human calcitriol (Vit. D) Western Blot analysis was used to quantified H3K9ac expression in BeWo cells after Vit. D stimulation.

**Figure 3:**
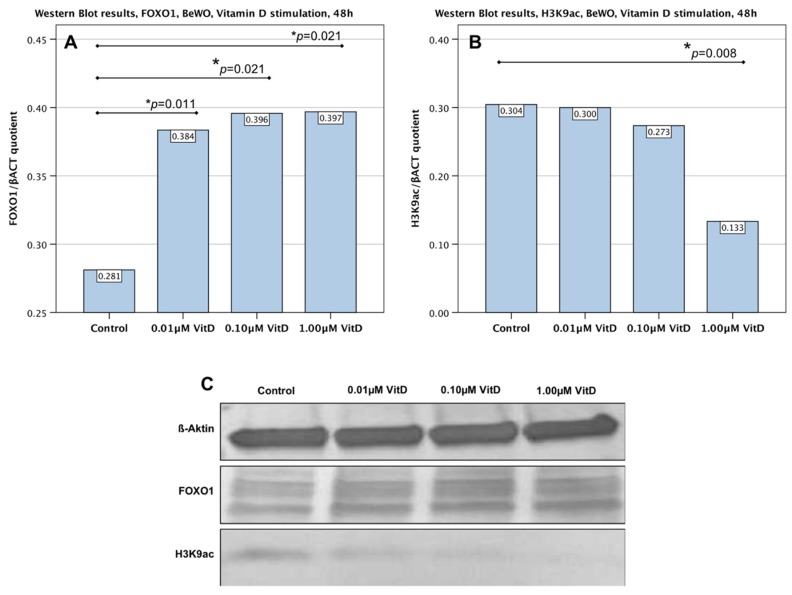
Western Blot analysis of FOXO1 and H3K9ac expression in BeWo cells after 48 h stimulation with human calcitriol. (**A**) Bar graph diagram showing a significant (*p* = 0.011 corresponding to 0.01 µM, *p* = 0.021 corresponding to 0.1 µM, *p* = 0.021 corresponding to 1.0 µM), dose-dependent upregulation of FOXO1 after 48 h stimulation with human calcitriol. (**B**) Bar graph diagram showing a significant (*p* = 0.008) downregulation of H3K9ac after 48 h stimulation with human calcitriol at 1.0 µM. No significance was shown for stimulation with 0.01 nor 0.1 µM. (**C**) Representative photographs of the western blot membrane with detected bands for H3K9, FOXO1 and endogenous control (β-Actin).

Quantitative Western Blot analysis showed that the 48 h in vitro culture with 1.0 µM human calcitriol resulted in a significantly lower H3K9ac expression in BeWo cells (Figure 3B,C; *p* = 0.008). However, the stimulation with lower concentrations of human calcitriol (0.1 and 0.01 µM), though lowering H3K9ac expression slightly, did not have a significant influence on the expression of H3K9ac (*p* = 0.515 for both concentrations). 

### 2.5. Upregulation of FOXO1 in Trophoblast Tumour Cells BeWo by Human Calcitriol (Vitamin D)

The expression of FOXO1 in BeWo cells after vitamin D stimulation was found to increase in a dose-dependent manner (Figure 3A,C). Statistical analysis showed the increased expression to be significant (*p* = 0.011) even after stimulation with the lowest concentration (0.01 µM). FOXO1 expression increased further with increasing concentrations of the stimulant, still showing statistical significance (*p* = 0.021).

### 2.6. Downregulation of H3K9ac in Primary Human Villous Trophoblast Cells HVT by Human Calcitriol (Vitamin D)

**Figure 4:**
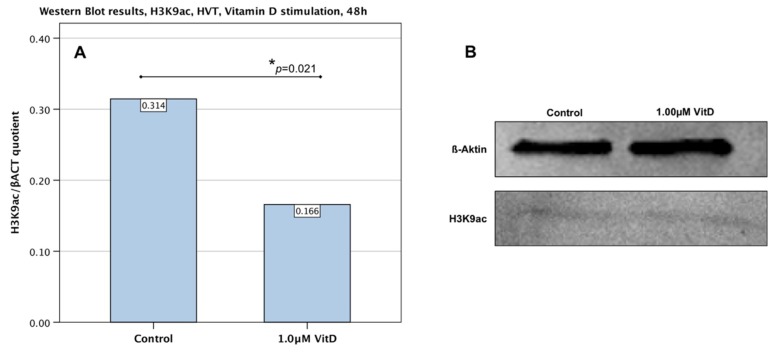
Western Blot analysis of H3K9ac expression in HVT cells after 48 h stimulation with human calcitriol. (**A**) Bar graph diagram showing a significant (*p* = 0.03) downregulation of H3K9ac after 48 h stimulation with human calcitriol at 1.0 µM. (**B**) Representative photograph of the western blot membrane with detected bands for H3K9 and endogenous control (β-Actin).

To solidify results concerning the downregulation of H3K9ac in BeWo cells, the experiment was repeated using human villous trophoblast cells. The stimulation of primary culture human villous trophoblast cells (HVT) with 1.0 µM human calcitriol showed an effect similar to BeWo cells. As seen in Figure 4, the expression of H3K9ac was significantly decreased (*p* = 0.03) after a 48 h incubation in comparison with the control culture.

## 3. Discussion

Dysregulation of histone modifications is an important factor in the pathophysiology of metabolic diseases and foetal programming, including GDM. The present study provides further evidence of this, as we identified a significant downregulation of H3K9ac in syncytiotrophoblast, EVT and foetal endothelial cells in GDM cases. Additional investigations of H3K4me3 did not show any dysregulation in GDM placentas.

While studies on insulin resistance were able to identify changes in histone modifications on a gene-specific level [46,47], this is the first study to show a pan-placental downregulation of H3K9ac in gestational diabetes mellitus. H3K9ac is an important modification for transcription activity in general and especially relevant for intrauterine development, synzytialisation and angiogenesis [26,48,49]. Animal studies have shown that treatment of somatic cell nuclear transfer embryos with histone deacetylase (HDAC)-inhibitors and the corresponding increase in global H3K9ac levels lead to improved embryo development and blastocyst quality [50]. Thus, the global H3K9ac downregulation found in GDM may indicate insufficient capacity of gene expression. This reduction in transcriptional activity could in turn be linked to foetal complications such as organ immaturity.

By now it has been well established that pregnancy-related diseases—including GDM—show sex-specific differences in terms of pathophysiology and outcome [51,52]. Very recently, Alexander, et al. [53] were able to show sex-specific differences in GDM on an epigenetic level. Thus, we considered a sex-disaggregated collection of data to be of great importance for the present study. The analysis of our data, however, did not show any sex-specific differences in H3K9ac expression.

Research investigating the “cross-talk” between H3K9ac and H3K4me3 indicates that acetylation of H3 tails is dependent on the recognition of and binding to H3K4me3. Thus, H3K4me3 could be seen as a docking basis for further activation of chromatin through H3 acetylation [54]. These findings correspond with the PTM’s localizations. As mentioned before, H3K4me3 is an essential part of the pre-initiation complex, forming the basis of transcription start, with transcription factor IID (TFIID) selectively binding to H3K4me3. H3K9ac is part of active enhancers, and as such potentiates transcription activity [55]. Taking this into account our findings may suggest that, while the basis for gene transcription is still intact, the activation of chromatin is in fact impaired in GDM placentas.

Investigating the role of histone modifications in insulin resistance, studies on beta-cells showed that treatment with incretin hormones, substances commonly used in treatment of DM2, leads to a global increase in H3K9ac. This in turn leads to an increase in cAMP response element binding protein/CREB regulated transcription coactivator 2 (CREB/CRTC2) transcription factor activity [56], indicating the functional relevance of H3K9ac. Since incretin generally improves insulin sensitivity, these findings suggest that low H3K9ac levels may contribute to insulin resistance. 

**Figure 5:**
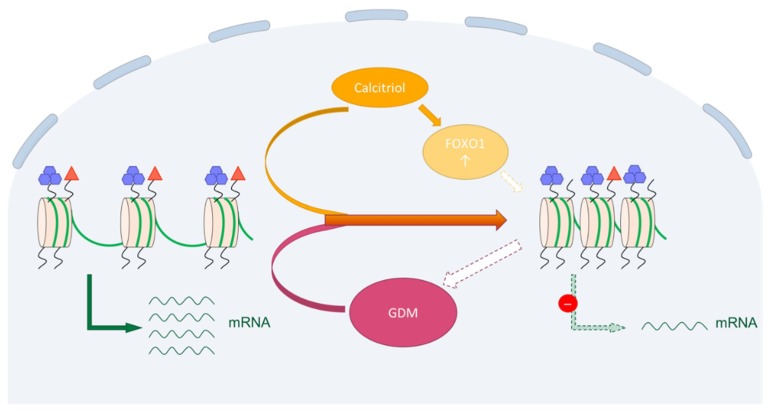
Two potential mechanisms leading to a reduction of H3K9ac in trophoblast cells. Immunohistochemical analysis showed a downregulation of H3K9ac (red triangle) in GDM placentas, while H3K4me3 (purple hexagons) showed no significant differences (solid pink arrow tail). The H3K9ac reduction may also be part of the aetiology of GDM (pink arrow broken line). Stimulation of trophoblast cells with calcitriol also lead to a reduction of H3K9ac, as well as an increase in FOXO1 (solid orange arrows). This may in turn be linked to the reduction of H3K9ac (yellow arrow broken line), ultimately leading to a downregulation of transcription (green arrows).

As outlined in the introduction, epidemiological as well as biochemical findings suggest a functional relationship between vitamin D, H3K9ac and FOXO1 with potential relevance for GDM. Thus, we hypothesized that treatment with vitamin D will positively affect H3K9ac (upregulate) and FOXO1 (downregulate). This hypothesis was tested through cell culture experiments using BeWo cells as a trophoblast model and HVT primary cultures to confirm (only H3K9ac). However, the results refuted our hypothesis. H3K9ac expression was not affected by low doses of calcitriol and decreased slightly at the highest concentration. FOXO1 expression increased after stimulation with calcitriol. While these results are consistent within each other, they resemble a state of glucose resistance rather than an improvement. Here it is important to note that stimulation of C2C12 muscle cells with calcitriol did result in a downregulation of FOXO1, suggesting that its role in metabolic control is highly cell type-specific [35]. We have summarized the immunohistochemical and cell culture findings graphically in Figure 5.

Extensive cell culture experiments using a dexamethasone as well as a TNF-induced model of insulin resistance were able to show that both agents induced VDR expression. Furthermore, they were able to show that VDR overexpression resulted in a reduction of insulin-mediated glucose uptake. These findings suggest that VDR is itself a mediator of different pathways of insulin resistance [57]. This may explain why treatment of BeWo cells with calcitriol, leading to an increased VDR activity, was unable to salvage H3K9ac and FOXO1 expression profiles, but rather mimicked a state of GDM. 

The fact that we found a dysregulation of H3K9ac not only in SCT and EVT, but also in foetal endothelial cells, may indicate long-lasting effects for the offspring in general and their vascular system in particular. There is evidence that even short-term exposure to hyperglycaemia induces lasting epigenetic changes in vascular cells [58]. These epigenetic changes were causally linked to changes in gene expression, leading to an inflammatory and proatherogenic state [59]. Taking this into account we hypothesis that the downregulation of H3K9ac in foetal endothelial cells may contribute to the foetal programming of cardiovascular disease associated with GDM. The role of H3K9ac in “metabolic memory” of the vascular system is controversial. Chen, et al. [60] showed an increase of non-specific H3 acetylation in human umbilical vein endothelial cells after glucose exposure. Furthermore, they found that the overexpression of p300, a histone acetyltransferase (HAT), results in similar expression profiles (e.g., transcription of VEGF-A and fibronectin) as glucose exposure. A global downregulation of H3K9ac was, however, found in endothelium and, in general, in a state of hypoxia [61,62], which in GDM may interact with the state of hyperglycaemia. Thus, further research is needed, concentrating on functional pathways associated with the global downregulation of H3K9ac.

In conclusion, our findings corroborate the growing evidence that epigenetic dysregulations play a key role in gestational diabetes mellitus. Due to the tremendous complexity of epigenetic mechanisms, conclusions concerning functional implications are to be drawn with extreme caution. The pan-placental downregulation of H3K9ac in GDM cases may, however, reflect a downregulation of transcriptional activity. Whether this is cause or effect of the metabolic disorder needs to be investigated further. Our cell culture experiments suggest that treatment with vitamin D is not sufficient to rescue the epigenetic and transcriptional changes in GDM, making this another area where further research is needed urgently.

## 4. Materials and Methods

### 4.1. Tissue Samples

The study design was approved by the Ludwig Maximilians University’s ethics committee and written consent was obtained from all participating patients. All participants underwent an oral glucose tolerance test (oGTT) between weeks 24 and 28 of their pregnancy. Using the criteria of the German society for Diabetes Mellitus (capillary whole blood; fasting glucose >90 mg/dL, 1 h > 180 mg/dL, and 2 h > 155 mg/dL) the diagnosis GDM was given when two measurements were above limits. For the study design, 40 patients with GDM and 40 healthy patients (control) were chosen to participate. In each group, foetal gender was balanced. For detailed information on clinical and epidemiological data see Table 1. All GDM patients underwent insulin treatment and were monitored at least once a week at the Diabetes Centre of the Department of Internal Medicine LMU Munich. The study was approved by the ethical committee of the University of Munich (project identification code: 337-06) on the 26th of August 2013 and informed consent was obtained from each patient in written form. Samples and clinical information were anonymized for statistical workup.

Tissue samples (2 × 2 × 2 cm^3^) of the participants’ placentas were obtain directly after birth. The pieces were taken from a cotyledon located the central part of placenta, with sufficient blood supply, aiming to contain decidua, villous as well as extra villous trophoblasts and amniotic epithelia. Areas showing signs of calcification, bleeding or ischemia were excluded from tissue collection. A 24 h incubation period in 4% buffered formalin solution served to fixate the tissue samples, after which they were embedded in paraffin for long-term storage.

### 4.2. Immunohistochemistry

#### 4.2.1. Staining

**Figure 6:**
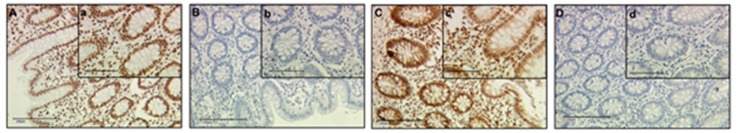
Positive and negative immunohistochemistry staining controls. Colon tissue was used as positive (**A**,**a**) as well as negative (**B**,**b**) control for H3K9ac antibody. For the H3K4me3 antibody, colon tissue was used as positive (**C**,**c**) as well as negative (**D**,**d**) control, as well. Negative control serum was added to the tissue for negative control staining. Brown staining indicates primary and secondary antibody binding, blue staining is due to haemalaun counter staining. Pictures were taken at a 100× (capital letters) and 250× (lower-case letters) magnification, respectively.

Immunohistochemical staining was carried out in accordance with the recently published protocol by Hutter, et al. [63]. After blocking the endogenous peroxidase using 3% H_2_O_2_, the slides were treated with sodium citrate (pH 6.0) in a high-pressure cooker in order to de-mask all protein epitopes. To prevent any unspecific antigen-antibody interaction, blocking solution was applied (ZytoChem Plus HRP Polymer System, Zytomed Systems GmBH, Berlin, Germany). The slides were then incubated with the primary antibodies (see Table 2) for 16 h at 4 °C. Antigen-antibody interaction was detected by applying the ZytoChem Plus HRP Polymer System (Zytomed Systems GmBH, Berlin, Germany) in accordance with the manufacturer’s instructions. Chromogen 3,3’-diaminobenzidine (DAB; Dako, Glostrup, Denmark) was used for staining followed by haemalaun for counterstaining. After dehydration, the slides were cover-slipped. For each experiment, positive and negative control staining was carried out on human colon tissue (Figure 6).

#### 4.2.2. Evaluation

Analysis of the tissue samples was conducted under a Leitz Diaplan microscope (Leitz, Wetzlar, Germany). The semi-quantitative *Immunoreactivity* Score (IRS) [64] was used to evaluate tissue staining. Multiplication of cell staining intensity (0: none; 1: weak; 2: moderate; 3: strong) with the percentage of positively stained cells (0: no staining; 1: <10% of the cells; 2: 11–50%; 3: 51–80%; 4: >81%) results in an IRS between 0 and 12 for each slide.

### 4.3. Double Immunofluorescence

To determine the phenotype of H3K9ac expressing cells in the placenta tissue, double immunofluorescence staining was performed. Placenta tissue of both the control and the GDM group were stained, using CK7 as a marker for EVTs and CD31 as a marker for foetal endothelial cells. 

Tissue samples from the same patient collective were used for double immunofluorescence staining as for immunohistochemistry. Pre-treatment of the slides (deparaffinising, blocking of endogenous peroxidase activity, de-masking of protein epitopes) was identical to that used for immunohistochemistry. Blocking solution (Ultra V–Block, Thermo Scientific, Lab Vision, Fremont, CA, USA) was applied for 15 min in order to prevent any unspecific antigen-antibody binding. Thereafter, the primary antibodies were mixed and applied together (see Table 2). Following this, the slides were incubated with the secondary antibodies (see Table 2) for 30 min. The slides were then cover-slipped with minimal light exposure using mounting buffer (Vector Laboratories, Burlingame, CA, USA), which contains DAPI for nuclear counterstaining. The phenotypes were analysed using the fluorescent Axioskop photomicroscope (Zeiss, Oberkochen, Germany). Images were taken with a digital Axiocam camera system (Zeiss, Oberkochen, German).

### 4.4. Cell Culture and Stimulation

The choriocarcinoma cell line BeWo (ECACC, Salisbury, UK) was used as a trophoblast model. Human villous trophoblast cells (HVT) (ScienCell, Carlsbad, CA, USA), a primary cell culture which was kryoconserved at −196 °C, was used to confirm BeWo results. Both cell lines were cultured in DMEM (3.7 g/L NaHCO_3_, 4.5 g/L d-glucose, 1.028 g/L stable glutamine, and Na-Pyruvate; Biochrom, Berlin, Germany) enriched with 10% foetal bovine serum (FBS) at 37 °C and 5% CO_2_. The BeWo as well as the HVT cells were grown in a 12-well plate at a density of 500,000 cells/mL DMEM for western blotting. To ensure adherence the cells were firstly cultured in DMEM with 10% FBS for 4 h, after which it was replaced with fresh DMEM not containing any supplementation. Following the 24 h incubation, the cells were stimulated with 0.01, 0.1 and 1.0 µM of human calcitriol (Sigma-Aldrich, St. Louis, MO, USA), dissolved in ethanol and diluted in DMEM without FBS. Corresponding amounts of ethanol were added to the control cells (see Table 3 for stimulation scheme). Stimulation lasted 48 h.

### 4.5. Westernblotting of Stimulated BeWo Cells and HVT Cell

The cells were treated with 200 µL of lysis buffer for 30 min, consisting of RIPA (Radioimmunoprecipitation assay buffer, Sigma-Aldrich, St. Louis, MO, USA) and protease inhibitor (Sigma-Aldrich, St. Louis, MO, USA) (dilution 1:100). The obtained lysates were centrifuged. To determine the protein concentration a Bradford assay was carried out. The amount of protein chosen for β-Actin and H3K9ac/FOXO1 detection were 5 and 20–25 µg respectively. Firstly, the samples’ proteins were separated by molecular weight through sodium dodecyl sulphate polyacrylamide gel electrophoresis (SDS-PAGE) and thereafter transferred onto a polyvinylidene fluoride (PVDF) membrane (Millipore, Billerica, MA, USA). Blocking of unspecific background staining was performed by incubating the membrane in 1× Casein solution (VECTASTAIN ABC-AmP Kit for rabbit IgG, Vector Laboratories, Burlingame, CA, USA) for 1 h. The primary antibodies, anti-H3K9ac (monoclonal rabbit IgG, Abcam, Cambridge, UK), anti-FOXO1 (monoclonal mouse IgG, Novus Biologicals Europe, Abingdon, UK) diluted at 1:500 in Casein and anti-β-Actin (monoclonal mouse IgG, Sigma-Aldrich, St. Louise, MO, USA) diluted at 1:1000 in Casein, were added for 16 h at 4 °C, plus an additional 2 × 15 min incubation at room temperature for anti-H3K9ac and anti-FOXO1. Following washing in 1× Casein solution the membrane was incubated with the respective secondary antibodies, biotinylated anti-rabbit-/mouse-IgG (VECTASTAIN ABC-AmP Kit for rabbit/mouse IgG, Vector Laboratories, Burlingame, MA, USA) as instructed by manufacturer’s manual. After 20 min treatment with ABC-AmP-reagent (VECTASTAIN ABC-AmP Kit for rabbit IgG, Vector Laboratories, Burlingame, MA, USA), the blots were developed with 5-bromo-4-chloro-3’-indolyphosphate/nitro-blue tetrazolium (BCIP/NBT)-chromogen substrate solution (VECTASTAIN ABC-AmP Kit for rabbit IgG, Vector Laboratories, Burlingame, MA, USA). Blots were detected using the Bio-Rad Universal Hood II (Bio-Rad Laboratories, Hercules, CA, USA) and quantitative analysis was performed using Bio-Rad Quantity One software (Bio-Rad Laboratories, Hercules, CA, USA).

### 4.6. Statistical Analysis

IBM SPSS Statistics for Windows, Version 22.0. Armonk, NY, USA: IBM Corp was used for data collection, data analysis and charts. More specifically, the non-parametric Mann-Whitney-U test was used for comparison of IRS results and the Wilcoxon-signed rank test for analysis of western blot results. P-values smaller than 0.05 were considered statistically significant.

## Figures and Tables

**Table 1:** Clinical and epidemiological data of study participants. The Kruskal–Wallis and Man-Whitney-U Tests were used for analysis of the data.

	GDM		Control		*p*-Value
	Male	Female	Male	Female	
Birthweight (g)	3662.1 ± 562	3635.9 ± 661	3339.8 ± 568	3294 ± 440	*p* = 0.019 *
Duration of gestation at delivery (weeks)	39.67 ± 1.30	39.83 ± 1.40	39.80 ± 1.54	39.75 ± 1.16	*p* = 0.943
Maternal BMI (pre-pregnancy)	29.38 ± 8.03	26.96 ± 4.73	21.92 ± 3.97	25.04 ± 7.90	*p* < 0.001 *
pH in umbilical artery	7.30 ± 0.07	7.30 ± 0.10	7.28 ± 0.10	7.30 ± 0.08	*p* = 0.826
Maternal Age (years)	31.46 ± 4.12	33.21 ± 5.33	30.30 ± 6.11	32.00 ± 6.13	*p* = 0.177
Vaginal delivery (%)	50	75	80	80	*p* = 0.207

Significant differences are marked with an asterisk (*).

**Table 2:** Antibodies applied for immunohistochemistry and double-immunofluorescence.

Antibody	Dilution	Incubation	Manufacturer
H3K4me3—polyclonal Rabbit IgG	1:500	16 h at 4 °C	Abcam—ab8580 (Cambridge, UK)
H3K9ac—Clone Y28 Rabbit IgG	1:200	16 h at 4 °C	Abcam—ab32129 (Cambridge, UK)
CK7—Clone OVTL Mouse IgG	1:30	16 h at 4 °C	Novocastra—NCL-L-CK7-OVTL (Newcastle, UK)
CD31—Clone JC/70A Mouse IgG	1:50	16 h at 4 °C	Abcam—ab9498 (Cambridge, UK)
Cy-2-labelled goat-anti-rabbit	1:100	30 min at RT	Dianova—115-226-062 (Hamburg, Germany)
Cy-3-labelled goat-anti-mouse	1:500	30 min at RT	Dianova—111-165-144 (Hamburg, Germany)

**Table 3:** Cell culture stimulation scheme.

**BeWo Stimulation for Western Blot (WB)**			
1.0 µM Ethanol	0.01 µM Vit. D	0.1 µM Vit. D	1.0 µM Vit. D
**HVT Stimulation for WB**			
1.0 µM Ethanol		1.0 µM Vit. D	

## Supplementary Materials

Supplementary materials can be found at http://www.mdpi.com/1422-0067/19/12/4061/s1. Figure S1: Sex-disaggregated H3K4me3 expression in syncytiotrophoblast (**A**) and decidua (**B**) of control and GDM placentas.

## Abbreviations

BMI	Body Mass Index
CD31	Cluster of differentiation 31
CK7	Cytokeratin 7
CREB/CRTC2	cAMP responsive element binding protein 1/CREB regulated transcriptional coactivator 2
DM2	Diabetes Mellitus Type 2
DMEM	Dulbecco’s modified Eagle’s medium
EVT	Extra-villous trophoblast cells
FBS	Fetal bovine serum
FOXO1	Forkhead box protein O1
g	Gramm
GDM	Gestational Diabetes Mellitus
h	Hours
H3K4me3	Trimethylation of Histone 3 at lysine 4
H3K9ac	Acetylation of Histone 3 at lysine 9
HAT	Histone acetyltransferase
HDAC	Histone deacetylase
HOMA-IR	Homeostatic model assessment—insulin resistance
HVT	Human villous trophoblast cells
IRS	Immunoreactivity score
LGA	Large for gestational age
M	Molar
mRNA	Messanger ribonuclease
ncRNA	Non-coding ribonuclease
oGTT	Oral glucose tolerance test
OR	Odds ratio
PTM	Post-translational modifications
RR	Relative Risk
SCT	Syncytiotrophoblast
TFIID	Transcription factor II D
TSS	Transcriptional start site
VDR	Vitamin D receptor
VEGF-A	Vascular endothelial growth factor A
Vit. D	Vitamin D
WB	Western Blot
