# Supplementary material for: Histone H3 Lysine 9 Acetylation is Downregulated in GDM Placentas and Calcitriol Supplementation Enhanced This Effect
Source: Int J Mol Sci. 2018 Dec 14;19(12):4061. doi: 10.3390/ijms19124061 (PMC6321349; doi:10.3390/ijms19124061)
Supplement: Supplementary file 1 [file ijms-19-04061-s001.pdf]

International Journal of
Molecular Sciences

MDPI

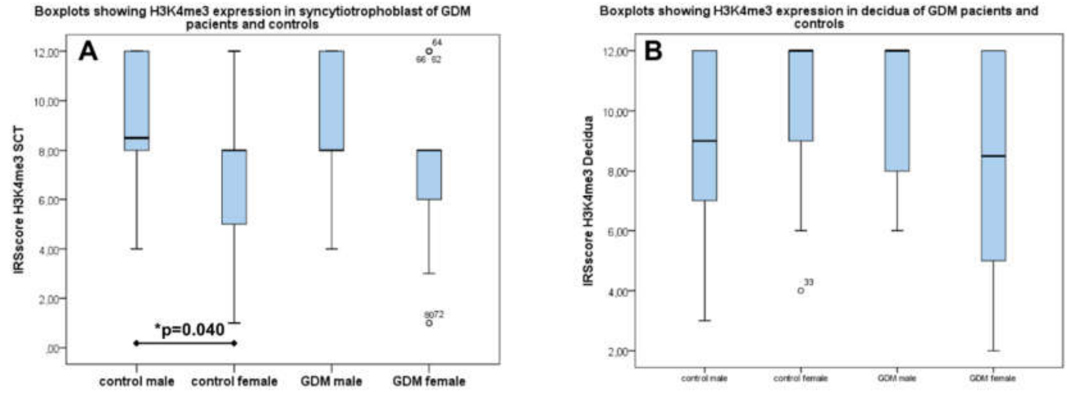

Figure S1. Sex-disaggregated H3K4me3 expression in syncytiotrophoblast (**A**) and decidua (**B**) of control and GDM placentas. Boxplots of the IRS show no significant differences in H3K4me3 expression. The syncytiotrophoblast of female control placentas displays a signigicantly lower (p = 0.040) H3K4me3 expression compared to male control placentas. The bars indicate the 5th and 95th percentiles. Circles indicate values more than 1.5-times the boxes' lengths.
